# Glaucoma

**Published:** 1884-09

**Authors:** F. R. Cross

**Affiliations:** Surgeon, Bristol Royal Infirmary and Bristol Eye Hospital; Consulting Ophthalmic Surgeon, Bristol Dispensary


THE BRISTOL

Medico-Chirurgical Journal.

SEPTEMBER, 1884.

### GLAUCOMA.

BY

F. R. Cross, M.B. Lond., F.R.C.S.,

*Surgeon, Bristol Royal Infirmary and Bristol Eye Hospital; Consulting Ophthalmic Surgeon, Bristol Dispensary.*

By extending Claud Bernard's experiment on the rabbit's ear, Loven has shewn (speaking in general terms) that stimulation of a sensory nerve is followed by a dilatation of the neighbouring blood vessels, and contraction of those of the rest of the body.

Irritation tends to increased local vascularity, and this to increase in the nutritive activity of the surrounding cellular elements; if the abnormal conditions continue, still further injurious stimulation follows, with spreading vaso-inhibition, blood stasis, and its results. When the inflamed centre lies in soft tissues, swelling and pus formation progress without much pain or irritation; but when it is situated in firm, unyielding tissue, as in bone, or under the periosteum, the tendency to swelling and exudation is prevented, the parts are severely compressed, and nerve worry, with further spreading vascular dilatation, follows. Pressure on the nerves produces agonising pain, high temperature, and severe constitutional disturbance, best expressed by the term *Tension Fever*. Locally there is an impairment of nutrition in the strangulated tissues, and, finally, their degeneration and death. Relief of the tension by incision through the infected nidus frees the nerves from irritation, and removes the cause of the constitutional condition; whilst the amount of local damage depends mainly on the intensity and the length of the period of strangulation. Large necrosis of a bone-shaft may result from unrelieved periostitis, but a complete recovery should rapidly follow an early incision.

The mischief done by tension must depend largely on the structure affected.

*Glaucoma* is a very serious disease, characterised by increased tension of the eye-ball.

Whilst the normal blood pressure supports a column of mercury 125 mm. in height, normal intraocular pressure supports 25 mm.; when 45 mm. is supported, eye-ball tension is definitely to be detected by palpation; but ocular pressure varies with the size and shape of the eye, the elasticity of the sclerotic, and the condition of the intraocular vessels. It is rarely increased in myopic eyes, though I have met with a few such cases.

The effects on sight of continued high intraocular pressure are so disastrous (sometimes most rapidly so, but more often insidiously, and by a sequence of symptoms whose importance is usually overlooked) that it would seem essential for every practitioner to be able to recognise the disease in its early stages, that proper treatment may be adopted before it is too late.

I endeavour to impress upon my students the extreme importance of learning to judge of intraocular tension, and of diagnosing both primary and secondary Glaucoma.

I fear that the experience of most ophthalmic surgeons would be that the sight in many *chronic glaucomas* is allowed gradually to fade away, and that in *acute* cases the importance of the eye in the train of symptoms is often overlooked.

*Acute Glaucoma* resembles inflammation. The symptoms of eye tension are very severe, their onset very sudden, with little tendency to spontaneous remission.

The chief complaint of the patient is neuralgia in the branches of the ophthalmic nerve, mainly around the orbit, with hemicrania, frequently accompanied by vomiting. The eye itself may be only slightly injected and dull-looking, with turgidity of the anterior ciliary veins, but usually it is intensely congested, with distensile pain increasing in severity; such cases are frequently mistaken for "bilious headache" or "rheumatic ophthalmia."

If attention is given to the eye, such a diagnosis is impossible: the cornea is dull, and the pupil, enlarged and sluggish, is pushed forward. Palpation shews that intraocular tension is very much increased, the eye-globe is quite firm and hard, vision is greatly impaired, and may be lost in a few hours. The second eye usually becomes affected in a like degree.

But destruction of sight follows eye-ball tension of far less severe type. Continuous or frequently-recurring pressure, of even slight degree, soon deteriorates the delicate elements of the retina and destroys their functions.

Between the most acute attacks, terminating in blindness within a few hours (*Glaucoma Fulminans*), and those in which sight gradually fails for a year or two, without any congestion of the eye or pain (*Glaucoma Simplex*), all kinds of intermediate cases are found; and most of them, if unrelieved, go on to blindness (*Absolute Glaucoma*).

The ordinary type is chronic, with subacute attacks of irritation periodically occurring. The eye is generally Hypermetropic, with a thick, unyielding globe. High tension on the *suspensory ligament* and *impaired ciliary action* are accompanied by rapidly-increasing Presbyopia, and *pressure on the retina* is expressed by its lowered function, dulness of sight, varying increase of cloudiness, especially after hard work, and progressive narrowing of the field of vision, especially in the lower part of the nasal side.

*Artificial lights* appear surrounded by haloes, red usually lying outside; in myopic eyes violet is outside.

*The Cornea* is more or less steamy, the *anterior chamber* shallowed by pushing forward of the iris, the *pupil* dilated, oval, and inactive; the *anterior ciliary veins* are congested and tortuous; there may be Scleral staphyloma.

*The media* are clear or more or less clouded; the *disc*, yielding most readily to the intraocular tension, is cupped; its vessels curve down over the edge, and both artery and vein pulsate; its central nerve-fibres suffer most, these pass to the periphery of the retina, and are concerned with the most peripheral parts of the field of vision, which is thus the earliest affected.

All these symptoms depend upon *increased pressure of the vitreous on its surroundings*.

Pain may be absent, or is dull and periorbital.

The early diagnosis of Glaucoma is most essential, not less so than that of acute inflammation in bone, or of strangulated hernia.

A very slight abnormal *pressure on the retina* is quite disastrous as regards sight, whilst a few hours' excessive intraocular tension will convert perfect vision into blindness.

And now as to the Pathology of the disease as a guide for treatment. The humours of the eye are secreted by the ciliary body; (*a*) the *aqueous* by the part lying in the posterior chamber, between the iris and the suspensory ligament; (*b*) the *vitreous* at the Zonule of Zinn by the posterior portion of the ciliary body where it lies on the Pars Ciliaris Retinæ.

The humours exude by exosmosis through many filters. Under normal conditions, pressure in the aqueous and vitreous are about equal; (*a*) the *aqueous* in the anterior chamber pushes back the iris upon the suspensory ligament (this acts like a valve, preventing regurgitation to the posterior chamber), and opens up the corneo-iritic angle, where it exudes through the trabeculae in the Ligamentum Pectinatum Iridis (Fontana's space) to Schlemm's canal and the anterior ciliary veins; (*b*) the *vitreous* filters, between the edge of the lens and the ciliary body, through the canal of Petit and the suspensory ligament, to the posterior chamber, where it joins the aqueous, and passes through the pupil.

If a diffusable liquid be injected into the aqueous, it escapes easily, and at a low pressure, along the tract mentioned; but injected into the vitreous, it does not escape, but pushes forward the lens, the ciliary body, and iris towards the cornea; closes the corneo-iritic angle, compresses Fontana's space (Lig. Pect. Iridis), and cannot escape.

Progressive obstruction to the outlet of the intraocular fluids at the corneo-iritic angle, with compression of Fontana's space, is probably always present in Glaucoma.

The Clinical Varieties of the disease depend on the nature of the morbid process causing the obstruction, or on its primary seat and character.

(*a*) *Changes of circulation or of nerve function*, especially in the ciliary body, or Pars Ciliaris Retinæ, may cause hypersecretion into the vitreous, with bulging forward of its anterior relations; or an altered character of the intra-ocular fluid may interfere with its normal tendency to diffuse through the suspensory ligament.

(*b*) *Swelling of the Ciliary Body* tends to obstruct Fontana's space in the ligamentum pectinatum iridis, or it may carry the adjacent iris forward, and narrow the corneo-iritic angle, or it may approach the edge of the lens and lessen the circumlental space.

(*c*) The perilenticular space is also narrowed by the *increased size of the lens*, which Priestly Smith has shewn to occur with advancing years, and to which he attributes the Glaucoma tendency of old people.

Hypermetropia *predisposes* to Glaucoma. The eye-globe is generally firm. The perilenticular space is narrowed by increased diameter and flattening of the lens, and by hypertrophy of the annular fibres of the ciliary muscle, described by Muller as the Compressor Lentis.

In a much narrowed circumlental space, or in a contracted corneo-iritic angle, complete obstruction might be readily excited.

*Exciting* causes are found under conditions of nervous depression, and these are associated with dilatation of the pupil; whereas premonitory attacks tend to subside when the pupil is contracted, as under warmth, food, and especially sleep.

The effect of dilatation of the pupil in exciting eye-ball tension, and of contraction of the pupil in reducing it, is attributed by Priestly Smith (to whose work on Glaucoma we are so much indebted) to the mechanical relations of the iris.

When the *pupil dilates,* the iris is gathered up in folds towards its ligament; this does no harm in the normal eye, but if the corneo-iritic angle is narrowed, and the iris pressed forward by the ciliary processes, complete blockage through Fontana's space in the ligament of the iris may result, and Glaucoma is established.

The converse occurs on *contraction of the pupil;* the iris is drawn from between the cornea and the ciliary processes, and the blockage is relieved.

It is well known, clinically, that eserin relieves eyeball tension, and that atropine has caused Glaucoma.

Some experiments by Höltzke, of Erlangen, showed that the aqueous pressure in cats' eyes increases with dilatation of the pupil, and falls with its contraction.

The lowest pressures recorded were under curare, or morphia. *Eserin increases the intraocular pressure considerably, and only lowers it when contraction of the pupil is established.*

Probably all mydriatics tend to increase Glaucoma, and all myotics to relieve it, in uncomplicated cases. Pilocarpin and curare might be used, but the action of eserin has been more studied ; a solution of two grains to the ounce should be put into the eye several times a day, in order to contract the pupil. During the premonitory stages its benefit is very marked on tension and on sight, but it seldom permanently cures Glaucoma; some predisposing cause is generally present which is readily excited to a repetition of the attack, or pathological changes may prevent relief of the obstruction at the filtration channel.

When eserin cannot contract the pupil, it is harmful; by its inhibitory effect on the blood vessels it increases the tension of the eye.

If Glaucoma is secondary to some other affection—and it may complicate almost any of the eye, especially where the ciliary body and the ligamentum pectinatum iridis are implicated—mydriatics (*Atropine, Duboisin, Daturin*) may be indicated. The rule should be, however, to use eserin in nearly all cases of Glaucoma until arrangements can be made for an operation, which is usually indicated without delay, in order to open up the obstruction to filtration, usually found at the periphery of the iris, between the ciliary body and the corneo-iritic angle.

1. *Simple paracentesis* affords temporary benefit. I have taken vitreous by an aseptic hypodermic syringe from eyes suffering from absolute Glaucoma, to the relief of pain. De Wecker uses a gold wire in such for continuous drainage.

2. *Cyclotomy or Myotomy* might be expected to modify the abnormal conditions existing in the ciliary body, and to be of value in acute cases, but probably no one would rely on it in these, whilst in chronic cases it can only relieve by forming a filtration scar by prolapse of the iris. I should never adopt it.

3. *Sclerotomy*, by dividing the periphery of the cornea, cuts near or through the main obstruction, but has hardly found favour with the majority of ophthalmic surgeons. De Wecker insists on the use of eserin before and after the operation, and specially warns against any bulging of the iris; Mr. Bader, on the other hand, encourages a permanent iritic staphyloma. Other details of the operation vary in the hands of different surgeons.

I perform sclerotomy in absolute Glaucoma, and on all my cases where the iris is much degenerated. The use of eserin widens the corneo-iritic angle, and renders it easier to pass the knife without entangling the obtruding iris. I make a long incision with a narrow sharp-pointed knife, having a short cutting edge, in this angle to a depth not quite reaching the subconjunctival tissue, and for about a fifth the length of the periphery of the cornea, taking great care to avoid prolapse of the iris, and use eserin frequently, afterwards. The operation relieves pain, but I am rarely satisfied with its permanent reduction of tension.

4. I have confidence only in a *large Iridectomy*, the corneal section made far back, so that the periphery of the muscle may be well removed, and without allowing any prolapse.

The sooner the operation is done the better; the amount of sight which the patient has retained up to the operation in *chronic* Glaucoma will usually be permanent, and may improve slightly for some months.

In *acute* cases its results are most brilliant; the abnormal symptoms disappear, and sight is restored more or less completely, according to the amount and duration of the abnormal pressure. Even though blind, the eye may almost perfectly recover after early iridectomy.

Of *malignant* Glaucoma I have no experience, and in the severe variety designated *Hæmorrhagic*, where the sclerosed vessels are liable to bleed into the vitreous or aqueous, and which follows retinitis apoplectica, I recommend no operation, as severe hæmorrhage or detachment of the retina might ensue, with sudden complete blindness.

